# AQ-MultiCal: An Interactive No-Code Machine Learning Platform for Low-Cost Air Quality Sensor Calibration and Comparative Model Analysis

**DOI:** 10.3390/s26082398

**Published:** 2026-04-14

**Authors:** Mehmet Taştan, Eren Cihan Karsu Asal, Hayrettin Gökozan

**Affiliations:** 1 Department of Electronics and Automation, Manisa Celal Bayar University, Manisa 45030, Turkey; 2 Department of Electric, Manisa Celal Bayar University, Manisa 45030, Turkey; eren.karsu@cbu.edu.tr (E.C.K.A.); hayrettin.gokozan@cbu.edu.tr (H.G.)

**Keywords:** low-cost sensors, sensor calibration, machine learning, hyperparameter optimization, AQ-MultiCal, healthcare engineering

## Abstract

The high installation and operational costs of reference-grade air quality monitoring systems have accelerated the widespread adoption of low-cost sensors (LCS). However, their susceptibility to environmental influences, temporal drift, and measurement uncertainty necessitates robust calibration approaches to ensure reliable measurements. Although machine learning (ML)-based calibration methods have been widely investigated, most existing implementations rely on static analytical workflows and require programming expertise, which limits their accessibility for many domain specialists. To simplify and standardize the calibration process for low-cost air quality sensors, this study presents Air Quality Multi-Model Calibration (AQ-MultiCal), an interactive, no-code platform. The platform provides a unified environment for evaluating 14 regression models, performing automated hyperparameter optimization, and conducting comparative performance analysis through an intuitive graphical interface supported by interactive visualization tools. The platform is validated using CO_2_ measurements collected from January and February 2025. Experimental results indicate that the optimized k-nearest neighbors (kNN) model achieved the best performance, with a coefficient of determination of R^2^ = 0.990 with low prediction error. These results demonstrate that AQ-MultiCal enables accurate sensor calibration and systematic comparison of ML models while improving the accessibility of ML-based calibration through an open-source platform designed for domain experts without programming expertise.

## 1. Introduction

Air quality is a critical environmental parameter due to its direct impact on human health and ecosystems. Anthropogenic activities such as industry, transportation, and agriculture have significantly intensified air pollution, making it a major global challenge [1]. According to the World Health Organization (WHO), approximately 90% of the world’s population is exposed to polluted air, contributing to nearly 7 million premature deaths each year [2]. Major air pollutants, including CO_2_, PM_2.5_, NO_2_, SO_2_, O_3_, and CO, pose substantial risks to both environmental sustainability and public health [3]. Consequently, continuous monitoring of air quality with high temporal and spatial resolution has become essential for effective environmental management and health protection [4].

Although reference-grade monitoring stations provide highly accurate measurements, their high installation and operational costs significantly limit their widespread deployment [5]. In addition, atmospheric variability and the need for specialized infrastructure further complicate large-scale air quality monitoring [6]. These limitations have motivated the development of LCSs, which offer a scalable and cost-effective solution for dense monitoring networks [7]. However, LCS measurements are inherently affected by environmental conditions, sensor drift, and nonlinear response behavior, which reduce their accuracy and long-term reliability [8,9]. Therefore, effective calibration techniques are essential to ensure data quality and enable reliable environmental assessments.

ML-based calibration methods have been widely adopted to address these challenges, particularly due to their ability to model complex nonlinear relationships between environmental variables and sensor responses [10,11,12,13]. Studies have shown that ensemble-based approaches and nonlinear models can significantly improve calibration performance under varying environmental conditions [14]. Furthermore, incorporating auxiliary meteorological variables and applying preprocessing techniques such as anomaly detection and signal smoothing have been reported to enhance predictive accuracy and long-term stability [15,16].

Despite these advancements, several limitations remain evident in the literature. Many studies focus on evaluating individual ML models or a limited subset of algorithms, which restricts systematic comparison across diverse modeling approaches and may hinder the identification of the most suitable models for different pollutants and deployment scenarios [17,18]. In addition, hyperparameter tuning is often performed using manually defined search strategies, which can reduce reproducibility and increase computational cost [19]. Calibration workflows are frequently implemented in programming-based environments, requiring manual coding for data preprocessing, model development, and evaluation, thereby limiting accessibility for domain experts without programming expertise [20]. Furthermore, many studies are designed for specific experimental setups and do not sufficiently address model generalizability across varying environmental conditions, locations, and deployment contexts [21]. Advanced approaches that incorporate uncertainty modeling or spatial analysis also face practical challenges due to their computational complexity and limited scalability [22].

In addition, although graphical user interface (GUI)-based ML tools and AutoML frameworks aim to simplify model development, they typically support a limited set of algorithms and lack integrated pipelines tailored specifically for sensor calibration and systematic model comparison. As a result, existing approaches remain fragmented and do not provide a unified analytical environment for comprehensive calibration analysis.

Therefore, a key gap in the literature is the lack of an integrated and user-accessible framework that enables systematic comparison of multiple ML models within a unified and reproducible calibration workflow. Existing studies predominantly focus on model-specific performance improvements or isolated calibration pipelines, rather than providing comprehensive and comparable analytical environments.

To address this gap, this study proposes AQ-MultiCal, an integrated, interactive, and no-code machine learning platform specifically designed for the calibration of low-cost air quality sensors. Unlike existing approaches that rely on fragmented workflows or programming-based implementations, AQ-MultiCal provides a unified analytical environment that integrates model selection, automated hyperparameter optimization, and comparative performance evaluation. The platform incorporates diverse machine learning models, including linear, tree-based, kernel-based, and ensemble learning approaches, enabling a comprehensive and systematic benchmarking environment. The platform enables the consistent evaluation of multiple regression models under identical conditions, thereby supporting more reliable and objective model selection. In addition, its user-friendly design improves accessibility for domain experts without programming expertise while enhancing reproducibility through a standardized workflow. Furthermore, the platform is publicly accessible, enabling its application across different datasets and environmental conditions.

This paper introduces AQ-MultiCal as a web-based, interactive, and no-code ML platform designed to streamline the calibration workflow of LCSs and address the limitations identified in existing studies. The system supports the systematic comparison of 14 regression models and incorporates automated hyperparameter optimization to improve model performance. Multiple optimization strategies, including grid search, randomized search, and Bayesian optimization, are supported to ensure flexible and efficient model tuning. In addition, controlled validation strategies are implemented to ensure robust and unbiased model evaluation. In addition, interactive visualization tools are provided to facilitate model interpretation and support data-driven decision-making. CO_2_ is selected as a representative pollutant to demonstrate the applicability of the proposed platform, while maintaining adaptability to other air quality parameters. By bridging the gap between advanced ML techniques and user-oriented environmental applications, AQ-MultiCal provides a scalable, accessible, and reproducible solution for large-scale air quality monitoring.

The main contributions of this study can be summarized as follows:

- Development of AQ-MultiCal, a unified and no-code ML platform for air quality sensor calibration;
- Design of an integrated framework enabling systematic comparison of multiple regression models;
- Implementation of automated hyperparameter optimization within a reproducible workflow;
- Integration of multiple optimization strategies (grid, randomized, and Bayesian search) for enhanced model tuning flexibility;
- Implementation of controlled validation mechanisms to ensure reliable and unbiased performance evaluation;
- Investigation of the effects of temporal, environmental, and dataset-related factors on calibration performance;
- Provision of interactive visualization tools to enhance interpretability and support decision-making.

The remainder of this paper is organized as follows. Section 2 presents the methodology and platform architecture. Section 3 describes the dataset and experimental results. Finally, Section 4 concludes the study and outlines future research directions.

## 2. Materials and Methods

### 2.1. Architecture of the Interactive Calibration Platform

AQ-MultiCal is a web-based, modular ML platform designed to support the complete calibration workflow, including configurable data splitting strategies, sampling interval selection, flexible model selection, and automated hyperparameter optimization. The system is implemented in Python using the Streamlit framework, providing an interactive, user-friendly interface for managing calibration processes. The architecture is designed to ensure scalability, reproducibility, and efficient handling of sensor data within a unified analytical environment. Within this architecture, the modeling process is formulated as an explicit input–output mapping. The input variables consist of raw measurements obtained from LCSs (e.g., CO_2_ concentration), along with optionally incorporated environmental parameters such as temperature and relative humidity. These inputs undergo feature scaling prior to being fed into the machine learning models. The model output corresponds to the calibrated sensor values aligned with the baseline measurement device. In this formulation, the model learns the nonlinear mapping between the low-cost sensor measurements and the baseline device observations, thereby minimizing systematic sensor errors. Consequently, the proposed approach frames the sensor calibration problem as a supervised learning task, aiming to improve measurement accuracy.

The developed platform is designed to operate on the Streamlit Cloud environment, providing researchers with a scalable and accessible interface. The platform integrates established ML libraries for model development and evaluation, together with efficient data-processing tools for handling large-scale sensor datasets. To enhance model interpretability and support scientific analysis, interactive visualization components are incorporated, enabling detailed examination of model performance, error distributions, and relationships among variables [23].

**Figure 1:**
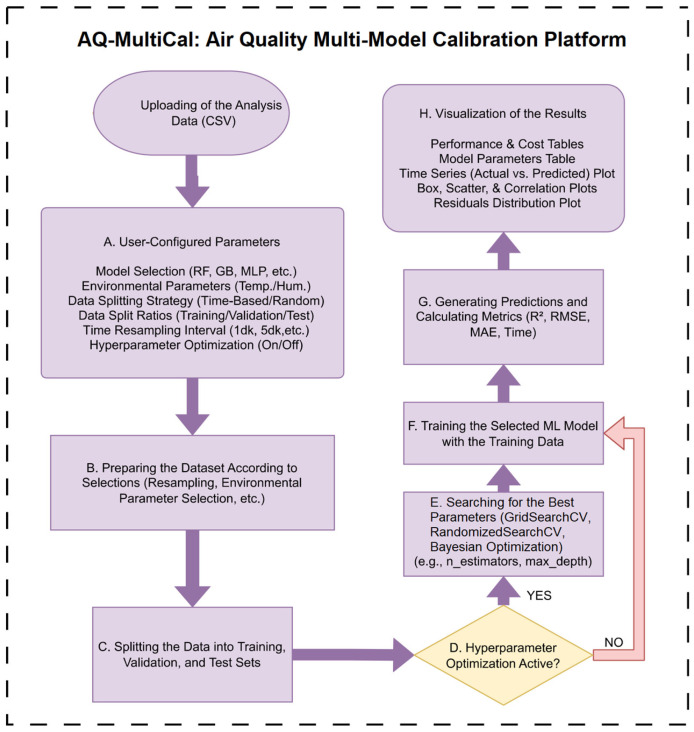
Workflow diagram of the AQ-MultiCal calibration framework.

The systematic workflow and architectural design of AQ-MultiCal, illustrated in Figure 1, are developed to bridge the methodological gap between complex ML operations and user-oriented environmental applications. This architecture enables researchers to manage advanced calibration workflows through an intuitive interface organized into four integrated functional layers.

The workflow begins with the data loading and configuration stage, where datasets are uploaded to the platform and prepared for analysis. Through an interactive control panel, users configure the calibration experiment by selecting one of the available 14 regression models and determining whether environmental parameters, such as temperature and relative humidity, should be included in the modeling process. To ensure reliable model validation, the platform then proceeds to the data partitioning and methodological configuration stage. At this stage, two different strategies are available: Random Splitting and Chronological (time-based) Splitting.

In the proposed framework, the dataset is divided into training, validation, and test subsets. When hyperparameter optimization is not enabled, the model is trained using only the training subset, while the validation and test subsets are used for performance monitoring and independent evaluation. When hyperparameter optimization is enabled, the training and validation subsets are combined and incorporated into a cross-validation procedure using a predefined split strategy to guide hyperparameter optimization. In this process, the test subset is kept entirely separate and is used solely for the final unbiased evaluation of the model on previously unseen data. This approach ensures a clear methodological separation between model selection and final performance assessment, thereby preventing data leakage.

The chronological strategy is particularly important for sensor calibration studies because it evaluates model performance by training on historical data and testing on subsequent observations, thereby mimicking real-world deployment scenarios. Following this configuration, the platform performs internal feature scaling using the StandardScaler method to eliminate differences in variable magnitudes between sensor measurements and environmental parameters. In addition, AQ-MultiCal incorporates a flexible hyperparameter optimization framework that supports Grid Search, Random Search, and Bayesian Optimization techniques. The optimization process can be executed either through an automated “Top 3 Parameters” configuration for computational efficiency or through a manual configuration that allows advanced users to define broader parameter ranges for detailed experimentation. In this study, the Grid Search strategy was employed for all analyses to ensure methodological consistency and reproducibility, thereby providing a standardized basis for objective comparisons among different sensors and regression models. In the final stage, the platform executes the calibration and optimization routines to evaluate model performance. The AQ-MultiCal platform enables not only the individual execution of different machine learning models but also the automated batch analysis of all supported models in a sequential manner; within this framework, performance metrics are computed for each model, and the results are systematically stored in the analysis history, thereby facilitating comparative performance evaluation across models on the same dataset and enabling data-driven selection of the most suitable model. The resulting outputs are presented through an interactive analytics and visualization layer that translates numerical results into interpretable scientific insights. Using the Plotly and Seaborn libraries, the platform generates dynamic scatter plots, residual density estimations (KDE), and a comprehensive set of performance metrics including R^2^, RMSE, MAE, and MAPE. This integrated analytical environment allows researchers to rapidly assess model accuracy, stability, and calibration performance, facilitating efficient data-driven decision-making in air quality monitoring applications.

### 2.2. Data Collection Process

The dataset used for calibration analyses was collected from five LCSs together with a commercial measurement device (Dienmern DM72b, Dienmern (Shenzhen) Science & Technology Co., Ltd., Shenzhen, China), which was used as a baseline instrument to provide reference measurements for comparative evaluation under co-location conditions. In addition, the CO_2_ sensor (MH-Z19B, Zhengzhou Winsen Electronics Technology Co., Ltd., Zhengzhou, China) integrated within the DM72b device is identical to the one used in the developed LCS units. This consistency reduces potential biases arising from hardware differences and enables the calibration models to focus on learning systematic discrepancies between sensor responses rather than absolute reference accuracy. Each LCS is integrated with an ESP8266-12E microcontroller (Ai-Thinker Technology Co., Ltd., Shenzhen, China), which enables wireless data transmission and real-time cloud connectivity, and is equipped with an MH-Z19B, a Plantower PMS7003 (PM_2.5_) (Beijing Plantower Co., Ltd., Beijing, China), and an AHT10 (temperature and humidity) sensor (Guangzhou Aosong Electronics Co., Ltd. (ASAIR), Guangzhou, China). These sensors had been deployed in various micro-environments (kitchen, bedroom, living room, bathroom, and outdoor) for a period of two years before the calibration campaign to expose the sensors to diverse real-world operating conditions and aging effects. The dedicated in-field data collection campaign was conducted during January and February 2025. To ensure a strictly homogeneous and controlled measurement environment during the calibration phase, the five LCSs and the baseline measurement device were co-located within a stabilized enclosure (25 × 30 × 40 cm), with sensors strategically positioned to minimize airflow-induced bias and inter-sensor interference, situated in a kitchen environment. This experimental setup was specifically selected to capture high-amplitude natural variations in CO_2_, temperature, and humidity, particularly those generated during indoor activities such as cooking, which are essential for robust model training. Data transmission was implemented using the Blynk IoT platform, which was used solely for real-time data acquisition and storage, at 15 s intervals and archived as 1 min temporal averages. Simultaneously, data from the baseline measurement device were synchronized at 1 min intervals via the Tuya integration platform, ensuring temporal alignment between sensor measurements. These platforms were utilized exclusively for data transmission and integration purposes and were not involved in the calibration or modeling processes. This operational phase yielded a high-resolution dataset comprising 84,960 paired observations for each parameter.

### 2.3. Data Preparation and Preprocessing

Before the modeling and optimization phase in AQ-MultiCal, raw sensor data underwent rigorous external preprocessing to ensure data integrity and synchronization with the baseline measurement device. Initially, timestamp synchronization and merging operations were executed between the LCS measurements and the baseline measurement data to establish a unified temporal framework. To enhance data quality, advanced techniques for missing-data imputation and signal cleaning were systematically applied in this external pipeline. Specifically, forward- and backward-fill and linear interpolation methods were used for univariate missingness, whereas kNN-based imputation was employed to address multivariate gaps by leveraging feature similarity across the dataset. This external process yielded a high-quality, standardized dataset for ML analysis by rectifying instrumental artifacts and inputting missing values. The resulting CSV dataset subsequently served as the standardized input for AQ-MultiCal, enabling feature scaling, model selection, and automated hyperparameter optimization within a unified framework. To facilitate evaluation and ensure scientific transparency, the benchmark datasets used in this study are publicly available via the AQ-MultiCal GitHub repository.

### 2.4. Platform Analysis Modules and User Interface

**Figure 2:**
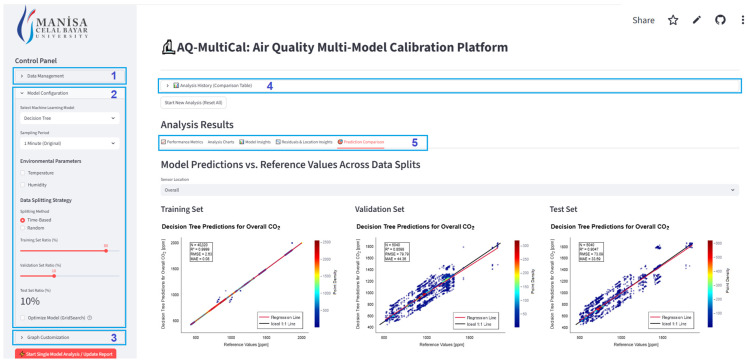
User Control Panel and Modules of the AQ-MultiCal Interface: (1) Data Management for uploading datasets; (2) Model Configuration for selecting algorithms and parameters; (3) Graphics Customization for interactive visualization settings; (4) Analysis History for tracking previous experiments; and (5) Analysis Results for comprehensive statistical performance assessment.

The architectural design and functional modules of AQ-MultiCal are illustrated in Figure 2. The system is engineered to democratize advanced ML workflows, allowing researchers to configure diverse analysis scenarios through a unified, no-code interface. The interface is partitioned into five primary functional segments: Data Management, Model Configuration, Graphics Customization, Analysis History, and Analysis Results.

In the Data Management (1) section, pre-processed, “analysis-ready” datasets are uploaded to the platform. The Model Configuration (2) section serves as the core decision-making hub, where users can select from 14 distinct ML regression models and determine the inclusion of auxiliary environmental parameters such as temperature and relative humidity. In this module, users define data splitting ratios for training, validation, and testing using either time-based or random splitting strategies. In addition, the platform integrates a hyperparameter optimization framework [24] that operates in both automatic and manual modes, providing a balance between computational efficiency and user control. The Graphics Customization (3) section facilitates the interactive exploration of results through dynamic visualizations, including residual distributions, time series, and correlation matrices. To ensure longitudinal tracking of experiments, the Analysis History (4) module enables the monitoring and comparison of results from previous or batch analyses. Finally, the Analysis Results (5) section provides a comprehensive quantitative evaluation of model accuracy using performance metrics such as R^2^, RMSE, MAE, and MAPE. This section also reports the computational cost (processing time in seconds) to evaluate the practical feasibility of each model. Advanced functionalities, such as Model Insights and Residuals and Location Insights, offer deeper diagnostic capabilities regarding model behavior and error patterns. To mitigate the risk of overfitting and improve the reliability of the calibration process, a cross-validation strategy is implemented within the platform. This approach ensures that the identified optimal hyperparameter sets are robust and not dependent on a single random data split. The platform’s model library encompasses a range of supervised learning approaches, including Linear Regression (LR), Ridge, Lasso, ElasticNet (EN), Stochastic Gradient Descent (SGD), RF, GB, AdaBoost (AdaB), kNN, DT, Multi-layer Perceptron (MLP), XGBoost (XGB), LightGBM (LGBM), and CatBoost (CatB).

Table flexible architecture of the platform allows the use of two distinct optimization modes based on user preferences. In the “Automatic Optimization” mode, the framework targets the hyperparameters highlighted in italic in Table 1, which are identified as the most influential parameters for each model [25]. This approach aims to improve predictive performance while maintaining computational efficiency. Conversely, the “Manual Optimization” mode grants users’ comprehensive control over the entire suite of parameters listed in the table, allowing for expert-level fine-tuning. To evaluate the predictive accuracy and generalizability of the models, standard regression performance metrics, including R^2^, RMSE, MAE, and MAPE, were employed. Among these, R^2^ is widely recognized as a highly informative metric for model evaluation [26]. These metrics assess the explained variance and error levels of each algorithm. Beyond statistical precision, the computational cost (analysis time in seconds) was also recorded to evaluate the computational efficiency and practical feasibility of the models.

The hardware and software environment utilized for these analyses is detailed in Table 2. The platform was developed using Python and leveraged core scientific libraries, including Scikit-learn for modeling, Pandas and NumPy for data manipulation, and Plotly and Streamlit for interactive visualization and interface deployment.

### 2.5. Hyperparameter Optimization

Hyperparameter optimization was performed using the GridSearchCV module from the Scikit-learn library. This process systematically searches for the combination of hyperparameters that yields the optimal performance for each model specified in Table 1. The parameter grids used for optimization were defined based on the characteristics of each model. For instance, for the RF model, the following parameter ranges were used: n_estimators [50, 100, 200, 300], max_depth [5, 10, 20, 30, None], and min_samples_split [2, 5, 10]. For the kNN model, the parameters n_neighbors [3, 5, 7, 11, 15, 20], weights [‘uniform’, ‘distance’], and metric [‘euclidean’, ‘manhattan’] were tested. The selected parameter ranges were defined based on commonly adopted values in literature and preliminary experiments to ensure a balance between computational cost and model performance.

The optimization was performed using the training data within a cross-validation framework. GridSearchCV evaluates each hyperparameter combination using cross-validation folds. This approach improves the reliability of performance estimates by preventing the models from overfitting a single data split. The optimal hyperparameter set was determined based on the R^2^ score as the primary optimization criterion in the GridSearchCV procedure, while RMSE was used solely as a complementary evaluation metric during cross-validation. In addition, other performance metrics such as MAE and MAPE were computed for comprehensive post-analysis and model comparison.

In this study, a k-fold cross-validation strategy (k = 5) was adopted to provide more robust and less biased performance estimation across different data partitions. In addition to Grid Search, the platform is designed to support alternative hyperparameter optimization strategies, including Random Search and Bayesian Optimization. These approaches provide flexibility in exploring the hyperparameter space, particularly for computationally expensive models or high-dimensional parameter configurations. However, in this study, Grid Search was exclusively employed to ensure methodological consistency, reproducibility, and comparability across models across all evaluated models.

## 3. Results and Discussion

This section presents a comprehensive evaluation of the developed platform’s effectiveness. The investigations are directed toward the impact of critical dataset parameters on model accuracy, the forecasting capabilities of various ML algorithms, and their respective computational requirements. The findings are discussed in light of the influences of environmental parameters and the efficacy of hyperparameter optimization. Furthermore, the cross-sensor consistency of the trained models and the practical efficacy of the platform are systematically evaluated across various experimental configurations. The developed platform is evaluated on the CO_2_ pollutant using fourteen different ML models.

The experimental matrix encompasses six sampling intervals (1, 5, 10, 15, 30, and 60 min), four data durations (1 week, 2 weeks, 1 month, and 2 months), and three data splitting ratios (60:20:20, 70:15:15, and 80:10:10). Additionally, the analysis integrates configurations involving environmental factors and automated hyperparameter optimization. This comparative framework aims to elucidate how diverse algorithmic architectures respond to variations in temporal resolution and to identify the optimal model for CO_2_ monitoring in terms of both statistical accuracy and computational efficiency.

### 3.1. Statistical Summary of the Dataset

The CO_2_ dataset, which constitutes the foundation of this study, undergoes a rigorous statistical characterization before calibration modeling. Following a two-year deployment in diverse micro-environments, all sensors are co-located with the baseline measurement device in a stabilized enclosure for a two-month calibration campaign within a kitchen environment. Consequently, the statistics presented in Table 3 characterize the performance and response profiles of each sensor under controlled and identical environmental conditions over the two-month period.

An analysis of Table 3 reveals distinct variations in measurement statistics among the sensors, despite their operation within an identical environment. These discrepancies are attributable to inherent differences in factory calibration sensitivity and temporal sensor drift induced by prolonged exposure. While the baseline measurement device recorded a mean concentration of 840 ppm, all LCSs consistently exhibited a positive bias by reporting higher CO_2_ levels. This finding underscores the imperative for ML-driven calibration to mitigate systematic biases and enhance measurement fidelity.

**Figure 3:**
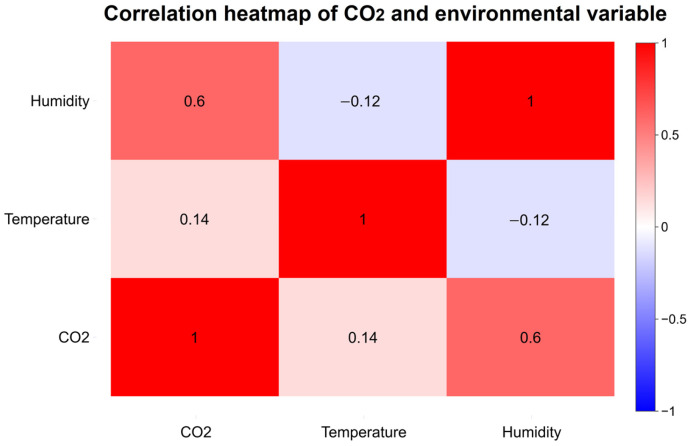
Inter-variable correlation heatmap illustrating the relationships between CO_2_ concentrations and environmental parameters.

The correlation relationships among the average data obtained from all sensors are visualized through the heatmap in Figure 3. According to this matrix, which illustrates the relationships between variables, a moderately positive correlation (0.6) is detected between CO_2_ and relative humidity. On the other hand, there is a weak positive correlation (0.14) between temperature and CO_2_. This correlation matrix provides important insights for evaluating the potential effects of feature selection and model performance during the training of ML models by highlighting the relative influence of environmental variables on sensor response behavior.

### 3.2. Effect of Data Parameters on Calibration Performance

**Figure 4:**
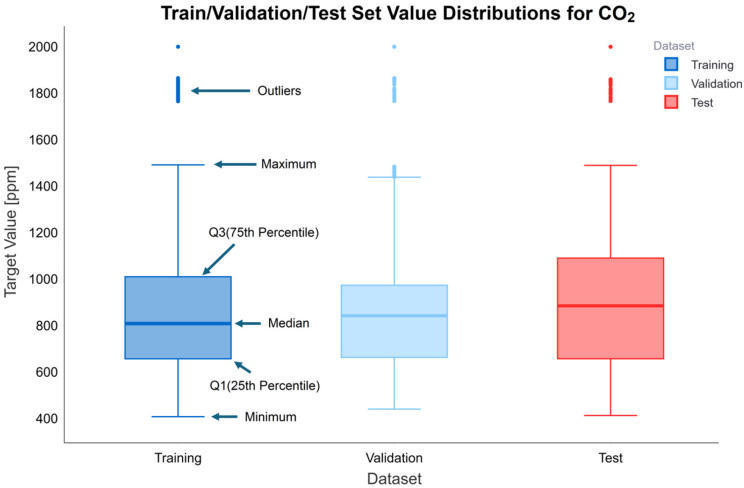
CO_2_ Concentration distribution in the training, validation, and test datasets.

In this section, the impact of critical dataset parameters on the performance of ML-based calibration models is evaluated. Accurate characterization and sub-setting of the data are essential prerequisites for the reliability and generalization ability of the models. For this purpose, the dataset is chronologically divided into training, validation, and test subsets using a time-based approach. The box plot in Figure 4 confirms that each subset corresponding to the 70:15:15 data splitting ratio has a similar statistical distribution. This balanced distribution is critically important for the models to exhibit consistent performance across different datasets.

The influence of the data sampling interval on the performance metrics is detailed in Table 4. The results indicate that optimal model performance for CO_2_ monitoring is attained at a high-resolution 1 min sampling frequency. This finding is consistent with previous studies showing that high temporal resolution improves predictive performance in air quality modeling [27,28]. These findings emphasize the importance of high temporal resolution data for accurately capturing dynamic air quality changes [29,30]. Specifically, the best performance for CO_2_ was obtained using the RF model at a 1 min sampling frequency R^2^ = 0.9864, RMSE = 31.2 ppm).

A pronounced degradation in performance metrics was observed as the sampling interval increased from 1 to 5 min. Consequently, the R^2^ value declined from 0.9864 to 0.8983 (an 8.9% decrease), while the RMSE increased from 31.2 ppm to 74.3 ppm, representing a substantial 138.1% escalation in error. In contrast, the performance metrics exhibited relative stability across the 5 to 60 min sampling intervals. Within this range, the R^2^ value experienced a marginal decline of 2.3% (dropping to 0.8773), while the RMSE showed a slight increase of 4.8%, reaching 77.9 ppm. These findings demonstrate that calibration efficacy is positively correlated with sampling frequency, whereas longer intervals lead to a loss of information and reduced predictive accuracy [19]. These results further demonstrate that tree-based ensembles, particularly RF, are more robust in capturing high-frequency temporal fluctuations due to their inherent ability to model complex nonlinear relationships without extensive feature engineering. Conversely, the performance degradation at lower sampling frequencies suggests that information loss adversely affects instance-based models like kNN, which rely heavily on local data structures. The analysis metrics reveal a distinct ‘bias-variance trade-off’ influenced by the partitioning strategy of training, validation, and test datasets [31]. In a static split, an excessively large training set may compromise the statistical reliability of validation and test metrics by disproportionately reducing their sample sizes. Conversely, increasing the proportion of the validation and test sets reduces the training data, which is critical for model learning, thereby degrading model performance [32].

In this study, to mitigate the limitations of static hold-out validation, “k-fold cross-validation” is preferred for hyperparameter optimization. The GridSearchCV tool utilized in the platform dynamically divides the training data internally into training and validation “folds”. This methodology eliminates the arbitrary effects of the data splitting ratio and ensures that the model’s performance is evaluated most robustly, without leaking into the “locked” test set. Comparative analyses of various data partitioning ratios, as detailed in Table 5, reveal that the 70:15:15 configuration yielded the optimal predictive performance for CO_2_ (R^2^ = 0.9864, RMSE = 31.2 ppm) [30]. The marginal performance variance observed across different splitting ratios underscores that the dataset is sufficiently comprehensive and representative of the underlying pollutant distributions. This lack of sensitivity to specific partitioning proportions serves as a key indicator of the models’ robust generalization capability. The generalization performance of sensor calibration models is fundamentally contingent upon the temporal duration of the training dataset [33]. Limited temporal durations, such as daily or weekly datasets, often induce overfitting to specific local conditions, thereby compromising model reliability during seasonal transitions or transient pollution peaks. Conversely, extended data durations, encompassing monthly or seasonal scales, expose the model to a broader operational spectrum, facilitating the learning of more robust and representative patterns [34].

Analysis of the varying data durations in Table 6 reveals a strong positive correlation between dataset duration and model performance metrics. Similar improvements with longer temporal data coverage have also been reported in air quality modeling studies [27]. Specifically, the R^2^ score for the kNN model improved from 0.9452 using a one-week dataset to 0.990 with a two-month dataset, marking a significant enhancement in predictive precision. Correspondingly, the RMSE exhibited a substantial reduction of approximately 52%, declining from 55.4 ppm to 26.8 ppm as the data duration extended to two months. This finding underscores the necessity of adequate data duration to effectively mitigate long-term performance degradation, such as sensor drift, while simultaneously strengthening the model’s generalization capacity [35]. From an ML perspective, extensive datasets enhance the representation of the underlying statistical distributions and minimize prediction variance, resulting in more stable and reproducible calibration performance.

### 3.3. Performance and Computational Cost Analysis of ML Models

This section provides a rigorous comparative analysis of the predictive performance and computational overhead of the ML algorithms integrated into the platform. Based on the 70:15:15 partitioning strategy, the empirical results in Table 7 indicate that non-linear architectures, specifically RF, DT, and kNN, exhibit superior predictive accuracy for CO_2_ monitoring. It is observed that non-linear models such as RF, DT, and kNN exhibit high prediction performance. The kNN model, after hyperparameter optimization performed on the training and validation subsets, achieved the highest predictive performance when evaluated on the independent test dataset, with an R^2^ value of 0.99 and an RMSE of 26.8 ppm [36]. The optimized RF model also yielded highly successful results, with an R^2^ value of 0.9886 [37,38]. This superior performance of the kNN model can be attributed to its instance-based learning structure, which effectively captures local patterns and non-linear relationships in the dataset, especially under high-resolution temporal data conditions.

Predictive performance analysis indicates a model hierarchy of kNN ≈ RF > DT >> GB ≈ LGBM ≈ CatB. Although kNN and RF reached the highest accuracy, the DT model showed competitive performance with substantially lower computational costs. In contrast, boosting-based algorithms, including GB and LGBM, exhibited lower performance under these specific dataset conditions. There are significant differences in the computational costs among these models. For instance, the optimized kNN model required 9.7 s for analysis, whereas the RF model took 4417 s to achieve similar accuracy levels. These results demonstrate the necessity of balancing predictive accuracy with computational efficiency during model selection. In this context, kNN emerges as a highly efficient model, offering the best trade-off between accuracy and computational cost, whereas RF provides slightly higher robustness at the expense of significantly increased computational time. These data clearly indicate that although different models can offer similar prediction accuracy, computational costs can vary significantly, especially in processes like hyperparameter optimization. This balance must be carefully managed according to project requirements. Considering that the training times for more complex models, such as MLP and DeepCNN, can take hours [39], the cost–benefit analysis feature provided by our platform is particularly noteworthy. Selecting models based on both accuracy and processing time is critical for real-time air quality monitoring in resource-limited applications. The stability of model performance across various configurations confirms the effectiveness of the platform in diverse calibration scenarios.

### 3.4. Effect of Environmental Factors

Environmental conditions influence the predictive performance of calibration models due to sensor response variations. The effect of including temperature and relative humidity on model accuracy was evaluated without hyperparameter optimization.

Table 8 shows that including environmental parameters reduced the performance of non-linear models, specifically RF, DT, and kNN [40]. For the DT model, the R^2^ value decreased by 10.1%, and the RMSE increased by 180%. These results indicate that unoptimized models, such as DT and RF, are susceptible to overfitting when the feature space expands without hyperparameter constraints [41]. This behavior is particularly evident when the feature space expands without corresponding constraints, such as depth limitation or minimum sample thresholds. The model memorizes the noise in the training data using these new environmental features, which dramatically increases the generalization error (such as the rise in RMSE) on the test data. Similarly, the performance of distance-based models like kNN is degraded due to the curse of dimensionality and the lack of feature scaling introduced by the added new features; features on different scales (CO_2_ vs. temperature) dominate the model’s “neighborhood” calculations, thereby weakening its predictive power. In contrast, including these parameters improved the performance of boosting (GB, CatB, LGBM) and linear (LR, Ridge, Lasso) models [11,42,43]. This improvement can be attributed to the regularization of mechanisms and the sequential learning structure of boosting algorithms, which allow them to selectively utilize informative features while suppressing noise. Similarly, linear models benefit from the additional explanatory variables when multicollinearity is controlled through regularization techniques.

### 3.5. Effect of Hyperparameter Optimization

The results in Table 7 show that hyperparameter optimization improves the performance metrics for CO_2_ calibration. The kNN model stood out as the most sensitive to optimization. Three-parameter optimization for kNN increased the R^2^ value by 0.96% and reduced the RMSE by 39%. Distance-based algorithms like kNN are sensitive to the scale of features (e.g., ppm vs. °C), and the n_neighbors parameter directly determines the model’s performance. On the other hand, the impact of optimization on computational cost is also noteworthy. Data in Table 7 indicate that the marginal performance gain from a third optimized parameter does not justify the increased computational cost. For the RF model, adding a third hyperparameter (min_samples_split) increased the analysis time from 1317 to 4417 s. These findings indicate that the balance between accuracy improvement and computational cost must be carefully considered during model optimization. These findings suggest the use of more efficient optimization methods, such as Randomized Search [44] or Bayesian Optimization [45], to reduce computational overhead.

### 3.6. Detailed Performance Analysis of the Best Models

This section evaluates the prediction consistency of kNN, RF, and DT models across sensors previously deployed in different micro-environments. Table 9 presents the performance metrics of the top three models for each CO_2_ sensor to evaluate the robustness of the calibration results.

All three models show high performance across all LCSs, with the kNN model achieving the highest accuracy for LCS1 (R^2^ = 0.9962, RMSE = 16.6 ppm). The results confirm that the platform captures the common characteristics of the sensors and provides stable corrections across LCSs, regardless of their prior environmental history.

**Figure 5:**
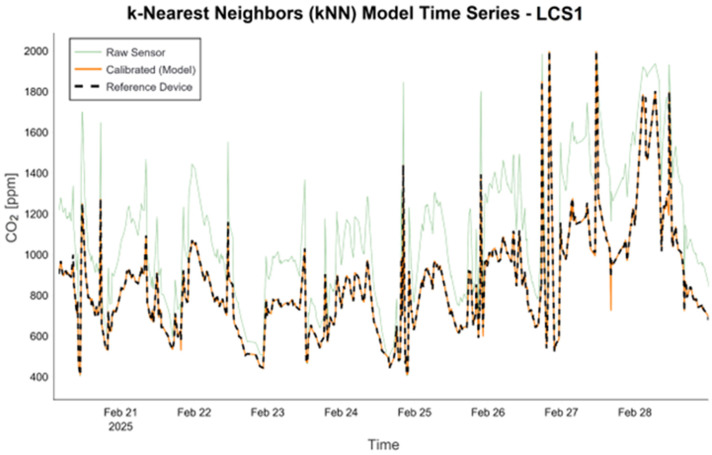
CO_2_ time series analysis for LCS1 with the kNN model.

Figure 5 presents a comparative time series analysis for the LCS1, highlighting the raw sensor data, kNN-calibrated predictions, and reference measurements. The plot demonstrates that the calibration model attenuates raw data fluctuations and aligns closely with reference values, thereby confirming the robustness of the time-dependent calibration.

**Figure 6:**
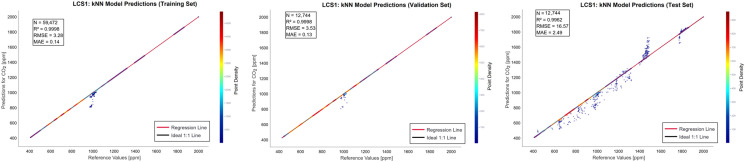
Prediction of the kNN model for LCS1.

The prediction distribution of the kNN model for the LCS1 is visualized in Figure 6. The proximity of the predicted values to the 1:1 line of parity, combined with a test set R^2^ of 0.9962 and RMSE of 16.57 ppm, demonstrates the robustness of the calibration process.

**Figure 7:**
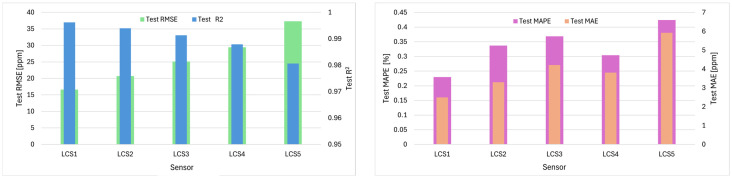
Performance metrics of the optimized kNN model across different LCSs.

The performance variations of the kNN model across various CO_2_ sensors are illustrated in Figure 7. While the LCS1 exhibited the highest performance (R^2^ = 0.9962 and RMSE = 16.6 ppm), the LCS5 showed the lowest (R^2^ = 0.9806 and RMSE = 37.3 ppm), suggesting that long-term exposure to different micro-environments impacts the measurement stability of identical sensors.

**Figure 8:**
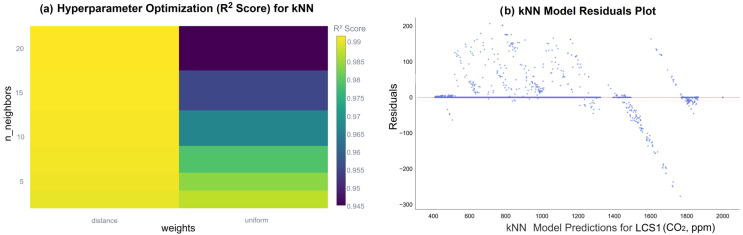
Optimization and error analysis for LCS1: (**a**) Impact of hyperparameter tuning on R^2^ performance, (**b**) residual distribution of the calibrated CO_2_ concentrations.

Figure 8a illustrates the impact of hyperparameter optimization on the R^2^ performance of the kNN model for LCS1. The analysis demonstrates that the optimization process led to a significant enhancement in prediction accuracy, particularly through the fine-tuning of hyperparameters such as n_neighbors and weights. This underscores the critical importance of hyperparameter selection in maximizing the calibration efficiency of non-linear algorithms like kNN. Furthermore, the residual distribution for the LCS1 CO_2_ concentration predictions, presented in Figure 8b, illustrates that the residuals are clustered around zero; this indicates that the model captures data patterns with minimal systematic bias.

**Figure 9:**
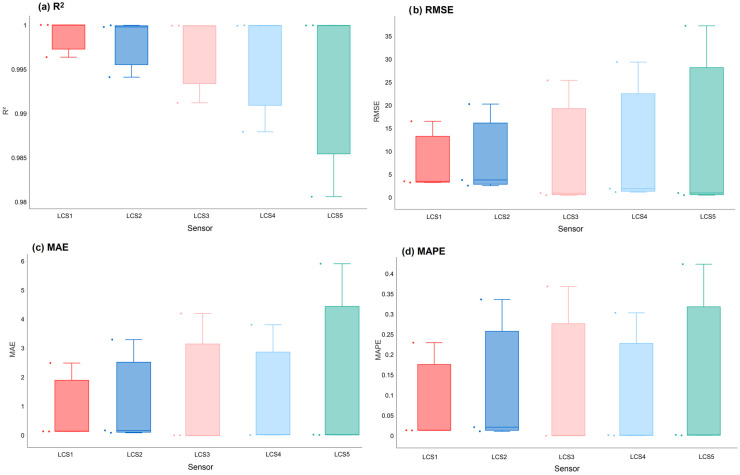
Performance metrics of the kNN model for the LCSs.

The statistical reliability and consistency of the kNN model across all LCSs are evaluated through the box plots presented in Figure 9. Analysis of the R^2^ values (Figure 9a) and error metrics, including RMSE (Figure 9b), MAE (Figure 9c), and MAPE (Figure 9d), reveals that the model maintains high predictive accuracy across diverse deployment scenarios. LCS1 exhibits the highest median R^2^ and the most compact distribution range among the LCSs. The high prediction performance of the kNN and RF models can be independently verified using the interactive tools provided by the AQ-MultiCal platform. Beyond the primary results focused on CO_2_, extensive analyses were conducted on PM2.5 data to further validate the cross-pollutant robustness of the AQ-MultiCal platform. Detailed performance metrics and computational cost evaluations for PM2.5 calibration are provided as Supplementary Materials (see Tables S1 and S2).

## 4. Conclusions

This study introduces AQ-MultiCal, an integrated, Python-based interactive ML platform designed to facilitate and streamline the calibration process of LCSs. The platform provides a unified interface that enables systematic sensor calibration for researchers and practitioners without requiring advanced programming skills. The proposed system significantly enhances research efficiency and ensures the reproducibility of analytical workflows by automating critical stages, including feature scaling, interactive data partitioning, and simultaneous model execution. Furthermore, AQ-MultiCal provides a robust hyperparameter optimization framework supporting Random, Grid, and Bayesian search strategies, offering users extensive flexibility for model fine-tuning.

The comprehensive analysis of a two-month dataset, spanning January and February 2025, yielded significant findings regarding the critical parameters and model behaviors that influence calibration performance. Non-linear regression techniques, specifically RF and kNN, demonstrated superior accuracy in the calibration of CO_2_ sensors. The optimized kNN model achieved an R^2^ of 0.990 and an RMSE of 26.8 ppm, providing a robust baseline for the dataset. The ability to simultaneously execute and evaluate 14 different regression models through the AQ-MultiCal framework established a rigorous empirical basis for identifying these optimal algorithms. The platform’s capacity to present analysis results through both comprehensive numerical metrics and diverse visual analytical tools—such as error distribution plots, scatter diagrams, and model comparison matrices—provides a significant advantage for researchers in interpreting complex outputs and making informed scientific decisions. The experimental results indicate that a 1 min sampling frequency and a 70:15:15 data splitting ratio are essential factors for model success. Furthermore, it was observed that the inclusion of environmental parameters had varying effects depending on the model architecture—improving the results for linear and boosting algorithms while reducing the performance of certain non-linear models. This highlights the necessity of conducting model-specific evaluations within an integrated environment to maintain data reliability across diverse monitoring scenarios. While hyperparameter optimization improved predictive accuracy, it also increased computational requirements; however, AQ-MultiCal addresses this trade-off by offering efficient execution times, typically under ten seconds.

Future research will prioritize the transition of AQ-MultiCal from a post-processing framework toward real-time calibration capabilities. A key objective is the integration of automated data cleaning and adaptive outlier detection modules, which will allow the platform to process raw sensor signals directly. Although the current architecture is pollutant-agnostic and supports various air quality parameters, its performance will be further validated through broader field deployments involving multi-pollutant sensor networks. By establishing these operational features, the platform aims to serve as a scalable, accessible, and autonomous decision-support tool for high-resolution environmental analytics. However, the use of a single dataset in this study is considered a limitation, and future work will focus on further validation using multiple datasets. In addition, a systematic evaluation of user experience and user satisfaction has not been conducted within the scope of this study. While the platform is designed as a no-code and user-friendly system, future work will include user-centered assessments to evaluate usability and practical adoption.

## Figures and Tables

**Table 1:** Optimizable hyperparameters for ML models.

Models	Optimized Hyperparameters
RF	*n_estimators, max_depth, min_samples_split*, min_samples_leaf, max_features, bootstrap
GB	*n_estimators, learning_rate, max_depth*, subsample, min_samples_leaf
kNN	*n_neighbors, weights, metric*, p
LR	*fit_intercept*
DT	*max_depth, min_samples_split, min_samples_leaf*, max_features
AdaB	*n_estimators, learning_rate*, *loss*, base_estimator__max_depth
SGD	*loss, penalty, alpha*, learning_rate, eta0
Ridge	*alpha, fit_intercept*
Lasso	*alpha, fit_intercept*
EN	*alpha, l1_ratio, fit_intercept*
MLP	*hidden_layer_sizes, activation, solver*, alpha, learning_rate_init
XGB	*n_estimators, learning_rate, max_depth*, subsample, colsample_bytree
LGBM	*n_estimators, learning_rate, num_leaves*, max_depth, subsample, colsample_bytree
CatB	*n_estimators, learning_rate, depth*, l2_leaf_reg

**Table 2:** Hardware and software environment where analyses were performed.

Category	Feature
Hardware	
Operating System	Microsoft Windows 10 Pro
Processor	Intel(R) Core (TM) i7-5500U CPU @ 2.40GHz (2 Cores, 4 Logical Processors)
RAM	16.0 GB
Software	
Programming Language	Python 3.12.5
Core Libraries	Pandas 2.2.2, NumPy 1.26.4, Scikit-learn 1.5.1, Plotly 5.22.0, Streamlit 1.37.1, Scikit-learn 1.5.1

**Table 3:** Comparative statistical metrics of CO_2_ concentrations for reference and LCSs.

Sensor/ Device	Deployment History	Count	Mean	Std	Min	0.25	0.50	0.75	Max
Reference	Calibration Box	84,960	840	251	408	659	798	976	2000
LCS1	Kitchen	84,960	1075	333	401	815	1033	1302	2000
LCS2	Bathroom	84,960	1304	306	404	1108	1312	1528	2000
LCS3	Livingroom	84,960	1259	295	405	1046	1267	1475	2000
LCS4	Bedroom	84,960	1118	331	404	874	1089	1338	2000
LCS5	Outdoor	84,960	1078	304	404	859	1058	1287	2000

**Table 4:** Effect of data sampling period on analysis metrics.

Model Name	Test R^2^	Test RMSE (ppm)	Test MAE (ppm)	Test MAPE (%)	Sampling Period (min)
RF	0.9864	31.2	7.04	0.63	1
GB	0.8983	74.3	65.5	6.98	5
MLP	0.8939	75.1	66.4	7.11	10
MLP	0.8920	75.6	66.5	7.18	15
GB	0.8807	76.6	67.3	7.29	30
Lasso	0.8773	77.9	68.5	7.46	60

**Table 5:** Effect of Data Splitting Ratios on Analysis Metrics.

Model Name	Test R^2^	Test RMSE (ppm)	Test MAE (ppm)	Test MAPE (%)	Train-Val-Test (%)
RF	0.9858	34.9	8.05	0.67	80:10:10
DT	0.9849	36.1	5.92	0.48	
kNN	0.9805	41.1	13.4	1.17	
RF	0.9864	31.2	7.04	0.63	70:15:15
DT	0.9855	32.3	5.12	0.44	
kNN	0.9806	37.3	12.2	1.17	
RF	0.9858	31.3	7.74	0.76	60:20:20
DT	0.9850	32.2	5.55	0.53	
kNN	0.9785	38.6	14.3	1.47	

**Table 6:** Effect of data duration on analysis metrics.

Model Name	Data Duration	Test R^2^	Test RMSE (ppm)	Test MAE (ppm)	Test MAPE (%)
kNN	1 week	0.9452	55.4	25.8	3.01
RF		0.9148	69.1	52.1	6.49
GB		0.9093	71.3	57.5	7.22
kNN	2 weeks	0.9803	32.8	9.7	1.29
DT		0.9706	40.1	11.6	1.52
RF		0.9678	41.9	20.7	2.74
kNN	1 month	0.9878	25.9	5.2	0.48
DT		0.9829	30.8	6.2	0.57
RF		0.9815	31.9	12.1	1.14
kNN	2 months	0.9900	26.8	3.95	0.33
RF		0.9886	28.6	5.55	0.48
DT		0.9878	29.6	4.10	0.35

**Table 7:** Model performance and computational cost table.

Model Name	Time (s)	Test R^2^	Test RMSE (ppm)	Test MAE (ppm)	Test MAPE (%)	Env. Factors	Optimized Parameters
GB	43.6	0.9376	66.9	54.4	6.35	Temp.	None
LGBM	7.91	0.9329	69.4	54.8	6.38	&	
CatB	73.2	0.9321	69.8	54.6	6.32	Hum.	
RF	67.7	0.9864	31.2	7.04	0.63	None	None
DT	3.00	0.9855	32.3	5.12	0.44		
kNN	1.93	0.9806	37.3	12.2	1.17		
kNN	10.6	0.9900	26.8	3.95	0.33	None	n_neighbors: 20, weights: distance
RF	1317	0.9886	28.6	5.55	0.48		n_estimators: 300, max_depth: None,
DT	15.7	0.9878	29.6	4.10	0.35		max_depth: None, min_samples_split: 2
kNN	9.7	0.9900	26.8	3.95	0.33	None	n_neighbors: 20, weights: distance, metric: euclidean
RF	4417	0.9886	28.6	5.55	0.48		n_estimators: 300, max_depth: None, min_samples_split: 2
DT	34.1	0.9878	29.6	4.10	0.35		max_depth: None, min_samples_split: 2, min_samples_leaf: 1

**Table 8:** Effect of environmental factors on model performance.

Model Name	Test R^2^	Chg (%)	Test RMSE (ppm)	Chg (%)	Model Name	Test R^2^	Chg (%)	Test RMSE (ppm)	Chg (%)	Env. Factors
DT	0.9855	−10.1	32.3	180	XGB	0.9203	0.83	75.6	−4.94	No
	0.8858		90.5			0.9280		71.9		Yes
kNN	0.9806	−7.9	37.3	124	AdaB	0.9183	−0.06	76.6	0.31	No
	0.9025		83.6			0.9178		76.8		Yes
RF	0.9864	−6.6	31.2	140	SGD	0.8365	1.09	108	−2.84	No
	0.9218		74.9			0.8456		105		Yes
GB	0.9263	1.2	72.7	−8	LR	0.8356	1.16	109	−2.99	No
	0.9376		66.9			0.8453		105		Yes
LGBM	0.9254	0.82	73.2	−5.19	Ridge	0.8356	1.16	108.6	−2.99	No
	0.9329		69.4			0.8453		105.4		Yes
CatB	0.9252	0.75	73.3	−4.77	Lasso	0.8353	1.22	108.7	−3.15	No
	0.9322		69.8			0.8455		105.3		Yes
MLP	0.9246	0.69	73.6	−4.30	EN	0.7250	1.18	140.5	−1.56	No
	0.9310		70.4			0.7335		138.3		Yes

**Table 9:** Performance metrics of the top three models for each CO_2_ sensor.

Sensor	Test R^2^	Test RMSE (ppm)	Test R^2^	Test RMSE (ppm)	Test R^2^	Test RMSE (ppm)
	kNN		RF		DT	
LCS1	0.9962	16.6	0.9950	18.9	0.9942	20.3
LCS2	0.9940	20.7	0.9918	24.3	0.9910	25.4
LCS3	0.9913	25.1	0.9892	27.9	0.9895	27.4
LCS4	0.9879	29.4	0.9877	29.7	0.9840	33.9
LCS5	0.9806	37.3	0.9793	38.5	0.9800	37.9

## Data Availability

The source code of the AQ-MultiCal platform developed in this study is publicly available on GitHub at https://github.com/tastan45/AQ-MultiCal, accessed on 5 April 2026. The interactive web-based application can also be accessed at https://aq-multical-73g6ufjhbbpplxpvza5efe.streamlit.app, accessed on 5 April 2026.

## Supplementary Materials

The following supporting information can be downloaded at: https://www.mdpi.com/article/10.3390/s26082398/s1, Table S1: Model performance and computational cost for PM2.5 data set-1; Table S2: Model performance and computational cost for PM2.5 data set-2.
