# Supplementary material for: AQ-MultiCal: An Interactive No-Code Machine Learning Platform for Low-Cost Air Quality Sensor Calibration and Comparative Model Analysis
Source: Sensors (Basel). 2026 Apr 14;26(8):2398. doi: 10.3390/s26082398 (PMC13119730; doi:10.3390/s26082398)
Supplement: Supplementary file 1 [file sensors-26-02398-s001.zip › sensors-4226302-supplementary.pdf]

This table presents the analysis results for $PM_{2.5}$ as an additional pollutant, separate from the primary focus of the manuscript. These results are provided to demonstrate the robustness and generalizability of the AQ-MultiCal platform and the calibration models across different air quality parameters.

DATA SET-1

Table S1. Model performance and computational cost for $PM_{2.5}$ data set-1.

Model Name	Analysis Time (s)	Test R^2	Test RMSE	Test MAE	Test MAPE(%)	Env. Factors	Optimized Parameters
GB	31.43	0.9482	2.30	1.84	11.31	Temp. Hum.	None
RF	63.03	0.9459	2.35	0.81	4.05		
Ridge	0.29	0.9444	2.38	1.95	13.23		
DT	1.28	0.9981	0.44	0.03	0.26	None	None
RF	33	0.9979	0.46	0.04	0.30		
kNN	3.21	0.9960	0.64	0.07	0.51		
RF	578	0.9983	0.41	0.03	0.27	None	n_estimators: 100, max_depth: None,
kNN	9.09	0.9980	0.46	0.03	0.32		n_neighbors: 20, weights: distance
DT	9.24	0.9979	0.46	0.04	0.30		max_depth: None, min_samples_split: 10
RF	1904	0.9980	0.45	0.04	0.30	None	n_estimators: 100, max_depth: None, min_samples_split: 5
kNN	15	0.9980	0.46	0.03	0.32		n_neighbors: 20, weights: distance, metric: euclidean
DT	12.4	0.9971	0.54	0.06	0.38		max_depth: None, min_samples_split: 2, min_samples_leaf: 4

DATA SET-2

Table S2. Model performance and computational cost for $PM_{2.5}$ data set-2.

Model Name	Analysis Time (s)	Test R^2	Test RMSE	Test MAE	Test MAPE	Env. Factors	Optimized Parameters
GB	29.10	0.98531	1.47	1.27	31.72	Tem. Hum.	None
CBoost	40.74	0.98507	1.48	1.03	25.29		
LGBM	2.37	0.98370	1.55	1.23	29.26		
RF	11.48	0.99761	0.59	0.15	20.09	None	None
DT	1.07	0.99757	0.60	0.15	20.08		
kNN	6.13	0.99258	1.04	0.25	38.91		
RF	321.03	0.99775	0.57	0.15	19.76	None	max_depth: None, n_estimators: 100
DT	2.62	0.99776	0.57	0.15	19.76		max_depth: None, min_samples_split: 5
kNN	13.79	0.99265	1.04	0.25	39.24		n_neighbors: 20, weights: distance
RF	921.03	0.99775	0.57	0.15	19.76	None	max_depth: None, min_samples_split: 5, n_estimators: 300
DT	5.66	0.99776	0.57	0.15	19.76		max_depth: None, min_samples_leaf: 1, min_samples_split: 5
kNN	23.07	0.99265	1.04	0.25	39.24		metric: euclidean, n_neighbors: 20, weights: distance
